# Genotype Meets Phenotype: Unraveling Gaucher’s Genetic Landscape in the Indian Population

**DOI:** 10.7759/cureus.92009

**Published:** 2025-09-10

**Authors:** Diana G Powline, Gnanapraba P, Tina Das, Suchanda Sahu, Sam M Joel, Praisy Joy

**Affiliations:** 1 Biochemistry, Madurai Medical College, Madurai, IND; 2 Biochemistry, All India Institute of Medical Sciences, Bhubaneswar, Bhubaneswar, IND; 3 Pediatric Surgery, Apollo Hospitals, Madurai, IND; 4 Anatomy, All India Institute of Medical Sciences, Bhubaneswar, Bhubaneswar, IND

**Keywords:** anemia, enzyme replacement therapy, gaucher’s disease, glucocerebrosidase, hepatomegaly, splenomegaly, thrombocytopenia

## Abstract

Gaucher disease is the most common autosomal recessive lysosomal storage disorder caused by mutations in the *GBA1* gene, leading to glucocerebrosidase deficiency and lipid accumulation in macrophages. In India, Gaucher disease poses a substantial public health issue among inborn errors of metabolism. This systematic review summarizes key clinical, genetic, and healthcare barriers of Gaucher disease in India. A total of nine Indian studies on Gaucher disease were systematically reviewed, integrating information on clinical presentations, diagnostic approaches, mutational landscape, treatment modalities, and survival trends. The review aims to detect consistent patterns and critical gaps in knowledge across distinct cohorts and geographic areas. Gaucher disease is the most prevalent lysosomal storage disorder reported among Indian cohorts, typically presenting with splenomegaly, hepatomegaly, anemia, and thrombocytopenia. The L444P mutation is the predominant genotypic variant observed. Enzyme replacement therapy improves survival, though access remains limited by cost. In India, Gaucher disease is marked by genotypic heterogeneity, dominated by the L444P variant, with outcomes depending on early and sustained treatment. Addressing diagnostic delays, infrastructure gaps, and cost barriers through national screening and registry systems is crucial.

## Introduction and background

**Figure 1:**
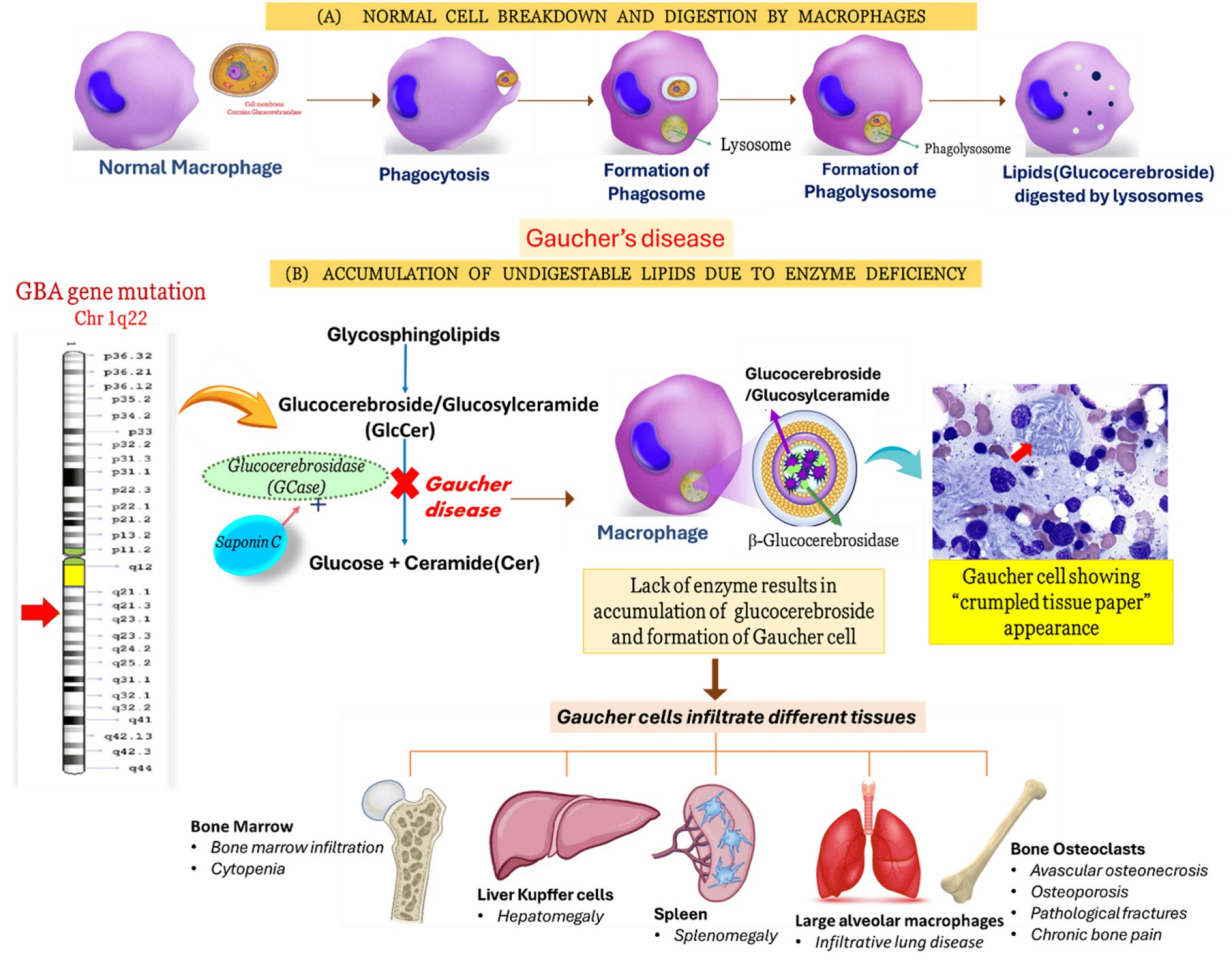
Pathophysiology of Gaucher disease. (A) Normal macrophages completely digest lipids (glucocerebroside) in lysosomes. (B) Hydrolysis of glucosylceramide (GlcCer) by glucocerebrosidase (GCase) in the lysosome. GCase is activated by saposin C. In lysosomal storage diseases, an enzyme deficiency results in the accumulation of its substrate in the cell lysosome (overload disease). In Gaucher disease, GCase deficiency results in an accumulation of GlcCer in the macrophages and formation of fibrillar aggregates in the cell cytoplasm, revealing a characteristic “crumpled tissue paper” appearance. These cells, known as Gaucher cells, infiltrate various organs (e.g., bone marrow, spleen, and liver) and are responsible for the major pathological signs of the disease. The figure has been modified from the original by the first author and reproduced with courtesy acknowledgement. Source: https://askhematologist.com/gauchers-disease/ [10].

Gaucher disease is an autosomal recessive lysosomal storage disorder, resulting from mutations in the *GBA1* gene. The *GBA1* gene is located on chromosome 1q21 and codes for the lysosomal glucocerebrosidase (GCase) enzyme, which cleaves the beta-glycosidic linkage of glucocerebroside lipids. Lysosomal GCase enzyme causes hydrolysis of glucosylceramide into ceramide and glucose, and *GBA1* gene mutation leads to a markedly reduced activity of the lysosomal GCase enzyme, leading to accumulation of glucosylceramide within the macrophage-lineage cells of the reticuloendothelial system (Figure 1) [1]. Very rarely, a defect in saposin C, causing a mutation in the *PSAP* gene, which is an activator of the GCase enzyme, can also lead to Gaucher disease [2]. Gaucher disease manifests with heterogeneous genotypic and phenotypic patterns due to the different degrees of severity involved in various organs [3]. Limited research in the Indian population has created a knowledge gap. Our review aims to address this gap by mapping genetic patterns and genotype-phenotype correlations in Gaucher disease.

History

Gaucher disease was described first by Philippe Gaucher in 1882 in a patient who presented with massive splenomegaly without leukemia. Gaucher detected large cells in a splenic aspirate while evaluating a case of splenomegaly, and he believed that it was evidence of a primary neoplasm of the spleen [4]. In 1924, Epstein first noticed the accumulation of glucocerebroside. Later, Brady et al. clarified that the accumulation was due to β-glucosidase enzyme deficiency [5].

Epidemiology

The global incidence of Gaucher disease is 1 in 40,000-60,000, with the Ashkenazi Jewish population showing a higher rate of 1 in 800 births [6,7]. A 2023 meta-analysis reported that the global prevalence of Gaucher disease was 1.5 cases per 100,000 live births [8].

Pathophysiology

The defective GCase leads to intracytoplasmic substrate deposition of glucosylceramide (GlcCer) in the macrophages, transforming these macrophages into Gaucher cells. The enlarged Gaucher cells have eccentric nuclei and condensed chromatin and cytoplasm and stain positive with periodic acid-Schiff, which gives the appearance of heterogeneous “crumpled tissue paper” under light microscopy (Figure 1B) [1]. Under electron microscopy, these GlcCer aggregates can be visualized as twisted fibrillar arrangements [9]. Gaucher cells in the macrophage-monocyte system, commonly seen in bone marrow, are the pathological hallmark of Gaucher disease.

Gaucher cells, originating from a distinct M2 macrophage subset with anti-inflammatory and regenerative function, cohabit with circulating M1 macrophages and induce a pseudo-inflammatory condition. Some molecules, such as chitotriosidase and CCL18, are expressed on Gaucher cells, which constitute specific disease biomarkers [11].

Liver and bone marrow biopsies show classic glycolipid-laden macrophages, especially in liver sinusoids. Hepatocytes remain largely unaffected due to their biliary excretion of glucocerebroside and exogenous glycolipid processing by mononuclear phagocytes, resulting in a low risk of hepatic failure. Osteoporosis is linked to inhibition of osteoblastic activity by interleukin (IL)-10, as well as to IL-1β, IL-6, macrophage inflammatory protein (MIP)-1α, macrophage colony-stimulating factor (M-CSF), and MIP-1β, which increases osteoclastic activity [12,13]. Histological assessment alone must not be used as a primary diagnostic tool.

Infiltration of Gaucher cells into the bone marrow, liver, spleen, and other organs leads to the disease symptoms. Infiltration of the bone marrow with Gaucher cells leads to vascular compression, which causes necrotic complications [14].

Classification

Gaucher disease has three phenotypic variants based on age and the presence of neurological deficits. Type 1, with no neurological damage, is the most common, whereas types 2 and 3 present with neurological impairment (Table 1). Neuropathic Gaucher disease represents a phenotypic continuum, which varies from the extrapyramidal syndrome in the mild form of type 1 to hydrops fetalis noted at the severe end of type 2 [15].

**Table 1:** Classification of Gaucher disease.

Feature	Type I (non-neuronopathic)	Type II (acute neuronopathic)	Type III (subacute/chronic neuronopathic)
Onset	Childhood to adulthood	Infancy (first 6 months)	Childhood or adolescence
Neurological involvement	Absent	Severe, rapid progression (e.g., bulbar palsy)	Present, slowly progressive (e.g., gaze palsy, dementia)
Visceral involvement	Present – splenomegaly, hepatomegaly	Severe visceral involvement	Present
Bone involvement	Common – bone pain, avascular necrosis, fractures	Absent	Present
Genetic mutation	N370S, others	L444P, complex alleles, recombinant alleles	Homozygous L444P or complex alleles
Diagnosis	Enzyme assay + genetic testing	Enzyme assay + genetic testing	Enzyme assay + genetic testing
Confirmatory test	↓Glucocerebrosidase activity in leukocytes + Gene sequencing	Same as type I + brain imaging + autopsy findings (if needed)	Same as type I + neurological exam, electroencephalography, MRI
Treatment	Enzyme replacement therapy (ERT) (imiglucerase, velaglucerase), substrate replacement therapy (miglustat), supportive	ERT not effective for central nervous system (CNS) symptoms; supportive only	ERT (limited CNS effect), supportive
Response to ERT	Good – improves blood counts, organomegaly, bone pain	Poor – no CNS improvement	Partial – visceral symptoms improve, CNS limited
Prognosis/Survival	Variable; normal life expectancy possible	Death before 2 years	Variable – can live to early adulthood or longer

Type 1 Gaucher Disease

Adult-onset (non-neuronopathic type) type 1 Gaucher disease presents with variable severity, including splenomegaly, hepatomegaly, and hepatic fibrosis, while hepatic failure and cirrhosis are rare [16]. Anemia and thrombocytopenia severity in patients with Gaucher disease correlates with splenectomy, with markedly higher hemoglobin and platelet levels seen post-splenectomy [17]. Skeletal findings often present as bone pain and sporadic painful episodes similar to vaso-occlusive crisis seen in sickle cell disease, most frequently in early-onset disease (<10 years). Characteristic features include osteolytic defects, vertebral collapse, pathological fractures, avascular necrosis, and Erlenmeyer flask deformity seen on distal femur imaging [17]. Growth retardation and delayed puberty are common, which show marked improvement with enzyme replacement therapy (ERT) [18]. GD1 shows links to akinetic-rigid Parkinsonism [19] and shows inconsistent genotype-phenotype correlation even between siblings and identical twins [20,21].

Type 2 Gaucher Disease

Acute neuronopathic Gaucher disease, also known as infantile cerebral Gaucher disease, is the rarest form, with an incidence of 1 in 150,000 live births. Type 2 Gaucher disease appears early in infancy with rapid neurological decline and extensive visceral involvement. It presents with eye movement abnormalities, bulbar signs, and severe visceral involvement, and is associated with a rapid neurologic decline, often proving fatal by nine months. Neuropathology reveals neuronal loss, gliosis, and Gaucher cell infiltration across multiple brain regions [21].

Type 3 Gaucher Disease

Subacute neuronopathic Gaucher disease has three subtypes. Subtype 3A causes ataxia and dementia, 3B affects multiple organs with gaze palsy, and 3C has ocular and cardiac involvement. Neurologic symptoms are variable and progress at different rates [21].

## Review

Methodology

Search Strategy

The search strategy for this systematic review was meticulously formulated according to the Preferred Reporting Items for Systematic Reviews and Meta-Analyses (PRISMA) 2020 guidelines [22]. We aimed to comprehensively aggregate and analyze studies focusing on genetic markers associated with Gaucher disease in the Indian population. To achieve an exhaustive collection of relevant literature, we conducted comprehensive searches across PubMed, Scopus, Web of Science, and Google Scholar for articles published from January 2005 to July 2025. We employed a combination of Medical Subject Headings (MeSH) terms and keywords that directly related to our research theme. These terms included “Gaucher Disease,” “Gaucher’s disease,” “glucocerebrosidase deficiency,” “GBA mutation,” “India,” and “Indian.” Boolean operators (“AND,” “OR”) were used to connect these terms strategically to optimize the search. To broaden the scope of our literature retrieval and ensure no relevant study was ignored, we additionally reviewed the bibliographic references of all included articles.

Eligibility Criteria

The eligibility criteria for this systematic review were systematically outlined to ensure the retrieval of the most pertinent and robust studies on genetic markers of Gaucher disease. We included original observational, cross-sectional, and cohort studies on Gaucher disease in the Indian population that reported genetic variants with confirmed *GBA1* mutations. Studies needed to provide clear data on genotype frequencies or associations between genetic variants, and needed to be published in the English language. Exposures covered common *GBA1 *mutations, such as N370S (N409S), L444P (L483P), D409H (D448H), RecNciI, or other complex alleles, identified by either β-glucocerebrosidase enzyme assay, *GBA1 *gene testing, or both. Comparisons were drawn across different mutation combinations, including homozygous versus compound heterozygous and mild versus severe mutations. Outcomes were focused on disease type, age of onset, symptom severity, organ involvement, bone and neurological features (in types 2 and 3), disease progression, and prognosis. To ensure an all-inclusive synthesis of contemporary evidence, our search timeframe extended from January 2005 to July 2025.

Studies that did not fulfil the inclusion criteria were excluded to uphold the focus and scientific integrity of the review. We excluded articles that were not peer-reviewed, review articles, editorials, conference abstracts, case series, case reports, and studies lacking genetic data to ensure reliability of our data sources. Studies on animal models or cell lines were also excluded. Moreover, non-English-language studies were excluded due to potential inaccurate translation affecting the interpretation of findings. This laborious selection process was designed to ensure that only the most relevant and high-quality studies were included in our systematic review, enabling a robust analysis of the genotype-phenotype correlation of Gaucher disease in the Indian population. Due to overlapping cohorts and heterogeneity in reported outcomes, quantitative pooling was not methodologically appropriate; hence, a systematic review approach was adopted.

Study Selection Process

**Figure 2:**
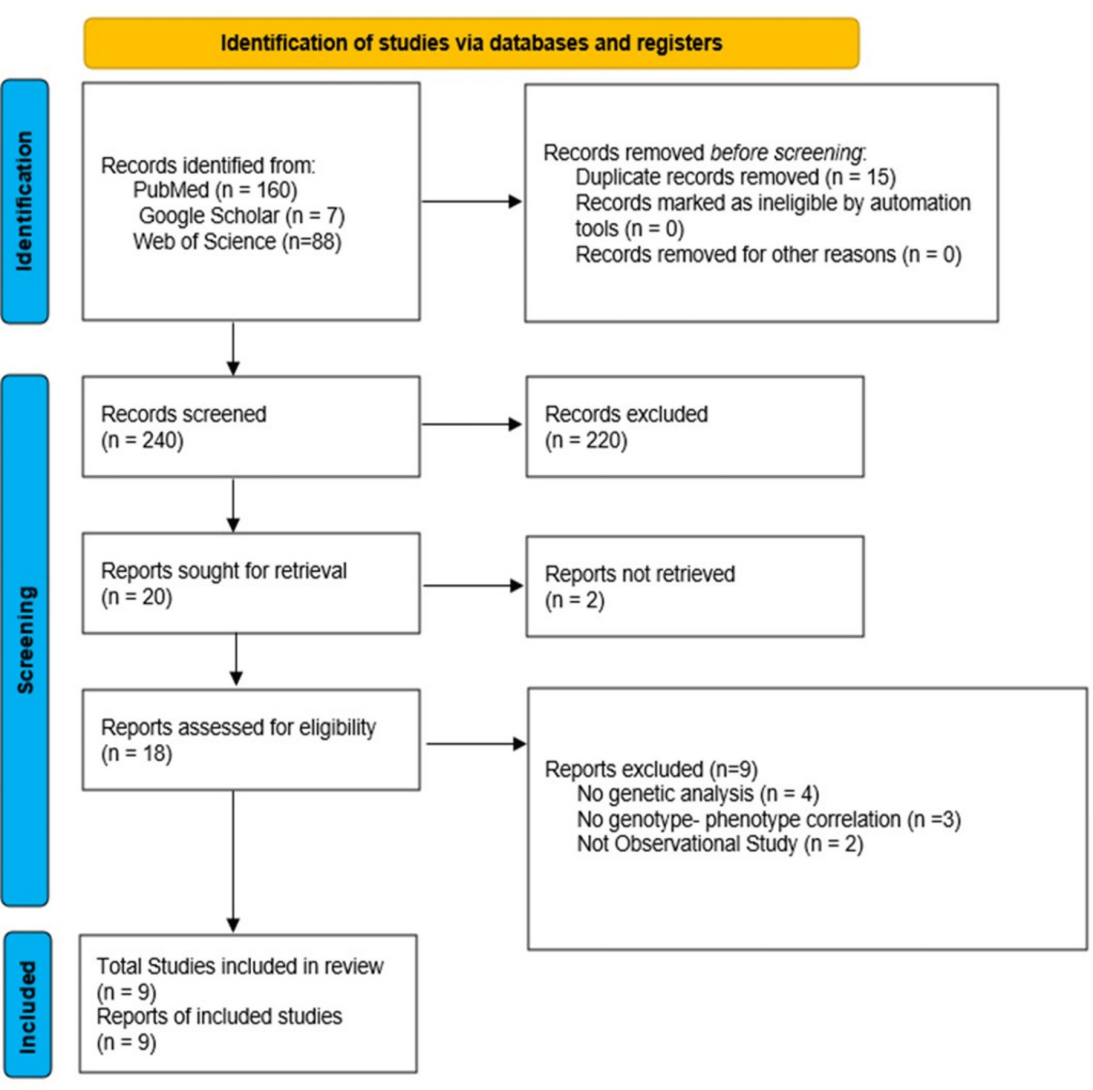
Preferred Reporting Items for Systematic Reviews and Meta-Analyses flowchart illustrating the selection process of reports included in this systematic review.

This review was conducted in accordance with PRISMA 2020 guidelines [22]. All identified search results were imported into Rayyan to delete duplicate articles and filter articles based on their titles and abstracts. Two reviewers independently screened the titles and abstracts, and the full texts of potentially relevant articles were retrieved and assessed against the predefined eligibility criteria. Discrepancies were resolved by census or discussion with a third reviewer. Ultimately, nine studies met all the requirements and were included in the review, providing a robust basis for our analysis of genotype-phenotype correlation among the Indian population. The overall study selection process is illustrated in the PRISMA flow diagram (Figure 2).

Data Extraction

The data extraction process was methodically designed to ensure precision and thoroughness. Articles were originally screened based on titles and abstracts to gauge relevance, with two independent reviewers categorizing them as “relevant,” “not relevant,” or “potentially relevant.” Articles considered “potentially relevant” were subjected to a full-text review using a predesigned Microsoft Excel form, allowing reliable and comprehensive data extraction based on predefined criteria. Discrepancies were resolved through discussion or third reviewer adjudication. The assessment was performed by three reviewers, and the fourth, fifth, and sixth reviewers revised the results. Data extraction captured core elements, including authorship, year of publication, study characteristics, sample size, genetic variants examined, phenotypic correlations, diagnostic methods, disease severity, response to treatment, and study limitations. This methodical approach ensured a comprehensive analysis, thereby strengthening the reliability of our findings in identifying the predominant regional genotypic variants and their phenotypic expression. The data extraction table has been included in the Appendices. The Joanna Briggs Institute Critical Appraisal Checklist for prevalence studies was used to evaluate the methodological quality of the included studies [23].

Data Synthesis

In this systematic review, we implemented a qualitative data analysis and synthesis approach due to the inherent heterogeneity of the studies reviewed. Our approach enabled a detailed evaluation of genotype variants associated with Gaucher disease across Indian cohorts and study designs. Data from each study were systematically categorized to recognize patterns, variations, and association strength. Our narrative synthesis combined these findings to present a comprehensive overview, noted consensus and gaps, while also assessing study quality and robustness. This approach yielded valuable insights into the genetic basis of Gaucher disease in the Indian population. Our synthesis aimed to provide a detailed panorama of genetic predispositions that contribute to Gaucher disease, thereby guiding future research directions and supporting therapeutic strategies.

Results

Demographic Characteristics

Across the nine included studies, 198 patients with confirmed Gaucher disease were analyzed. The studies spanned retrospective clinical evaluations, enzymatic diagnosis, and molecular profiling, offering a comprehensive overview of Gaucher disease in Indian cohorts (Table 2). The age at presentation ranged from infancy to the third decade of life, with the majority of patients presenting during early childhood (mean age = 7.8 years; range = 6 months to 24 years) [24-26]. Male predominance was observed in most studies, with a pooled male-to-female ratio of approximately 1.6:1 [25,27]. A high rate of consanguinity was reported in some regions, particularly in southern and eastern India [24,26].

**Table 2:** Demographic characteristics of Gaucher disease patients in Indian studies (n = 9). LSD = lysosomal storage disorder

Authors	Total participants	Gaucher disease patients	Age range (years)	Sex distribution (male/female)	Region/Ethnicity
Verma et al. [28]	444 suspected LSD	23 (5.2%)	0.4–15	14/9	North India; a tertiary genetics center
Ankleshwaria et al. [21]	33	33	0.5–36	18/15	Pan-India
Muranjan and Patil [29]	37	37	Onset: 0.1–12.5	22/15	Western India
Sheth et al. [30]	7	7	20–40	2/5	Gujarat, Punjab, Uttar Pradesh, Maharashtra
Sheth et al. [31]	100	100	0.1–45	62/38	Pan-India; East = 4%, West = 43%, North = 32%, South = 21%
Barney et al. [25]	60	60	0.17–40 (median: 2 years)	33/27	South India (Christian Medical College Vellore)
Goyal et al. [27]	21 LSD patients	7	0.3–12	5/2	North India
Magar et al. [26]	22 screened	9	0.03–2 (12 days to 24 months)	6/3	A pediatric hospital
Sheth et al. [24]	1,200	69	0.2–42	38/31	Pan-India; FRIGE Ahmedabad

Genetic Mutation Spectrum

The most common mutation across all studies was p.Leu483Pro (L444P), seen in more than 60-70% of Gaucher disease patients, followed by RecNcil, p.Arg535Cys, and p.Asn409Ser. Several novel and rare mutations were reported, some of which were compound heterozygotes or large deletions (Table 3).

**Table 3:** Common and novel GBA1 gene mutations in Indian Gaucher disease (GD) patients.

Mutation	Amino acid change/Exon location	Frequency	Associated GD type(s)	Notes	Studies
c.1448T>C missense	p.Leu483Pro (L444P) exon 10	60–70%	Types 1, 3	Most common	Sheth et al. [31], Ankleshwaria et al. [21], Barney et al. [25]
RecNcil complex	Complex allele Exon 9-10	~7%	Types 1, 2	Severe phenotype	Sheth et al. [31]
c.1504C>T missense	p.Arg535Cys Exon 11	~3–5%	Type 1	Common	Sheth et al. [31], Barney et al. [25]
c.1226A>G missense	p.Asn409Ser N370S	3%	Type 1	Mild GD1 form	Multiple
Missense	p.Gly289Ala	2%	Type 1 or 3	Likely pathogenic	Ankleshwaria et al. [21]
Missense	p.Ile466Ser	2%	Type 1 or 3	Likely Pathogenic	Ankleshwaria et al. [21]
Missense	p.Gly383Asp, p.Gly399Arg	2%	Type 1 or 3	Pathogenic	Multiple
Deletion	Exon 4–10	Rare	Type 2/3	Confirmed by MLPA	Barney et al. [25]
Compound heterogeneous recombinant allele from a pseudogene	L444P/RecNcil exons 9–10	Rare	Type 2	Seen in severe or neuronopathic GD	Sheth et al. [31], Barney et al. [25]

Clinical Characteristics

Most patients presented with hepatosplenomegaly, anemia, bone pain, and neurological symptoms in type 2 or 3 Gaucher disease. Age at diagnosis varied from infancy to late childhood. Enzyme assay, molecular sequencing, and bone marrow findings were the primary diagnostic tools (Table 4).

**Table 4:** Clinical characteristics of Gaucher disease (GD) patients in Indian studies. LSD = lysosomal storage disorder; HSCT = hematopoietic stem cell transplantation

GD type	N	Age at diagnosis	Common features	Diagnostic tests	Notes	Studies
Type 1 (mostly)	100	-	Hepatosplenomegaly, cytopenia	Enzyme + sequencing	High mutation detection rate	Sheth et al. [31]
Type 1	33	3 years (med)	Bone pain, marrow infiltration	Enzyme + polymerase chain reaction	Novel variants reported	Ankleshwaria et al. [21]
	9/22	Pediatric	Thrombocytopenia	Dried blood spot	Screening utility	Magar et al. [26]
All types	60	2 years (med)	GD1: cytopenia; GD3: neurological signs	Enzyme + marrow + DNA analysis	HSCT outcomes presented	Barney et al. [25]
Mixed GD	224	-	Registry data	Enzyme-based	Large-scale burden estimate	Sheth et al. [24]
GD1/2/3	10	6 months to 7 years	Growth delay, hepatosplenomegaly	Enzyme (blood/fibroblast) + genetics	Prenatal diagnosis in 1 GD family	Verma et al. [28]
GD not confirmed	32	-	Classical LSD/GD features	Enzyme assay or genetic confirmation	Combined data used	Three additional studies [26,27,29]

Therapeutic Approaches and Outcomes

Therapy modalities included ERT, substrate reduction therapy (SRT), and hematopoietic stem cell transplantation (HSCT) (Table 5). ERT was initiated in multiple centers, with favorable hematological and organ responses. Barney et al. reported significantly better survival in patients receiving definitive therapy [25].

**Table 5:** Treatment modalities and outcomes in Indian GD patients. ERT = enzyme replacement therapy; HSCT = hematopoietic stem cell transplantation

N	Treatment modalities	Mortality rate	Notes	Studies
60	ERT (9), HSCT (3), supportive care	10% (definitive) vs. 47.5%	Kaplan-Meier analysis showed a significant survival difference	Barney et al. [25]
1	ERT	—	Platelet count and hemoglobin improved	Magar et al. [26]
4	ERT (GD1 cases)	Not specified	Liver/spleen size and growth improved	Verma et al. [28]
Various	ERT, supportive, prenatal diagnosis	NA	Varies per institutional access	Others [24,27,30,31]

Discussion

Gaucher disease, the most common lysosomal storage disorder caused by *GBA* gene mutations and subsequent glucocerebrosidase enzyme deficiency, poses a significant healthcare burden in India. Indian studies on Gaucher disease reveal a distinct genotypic pattern with profound clinical heterogeneity. Regional data frequently identify Gaucher disease as the most prevalent lysosomal storage disorder in India. For instance, a retrospective study from Rajasthan reported Gaucher disease in 46.1% of pediatric lysosomal storage disorder cases [28], and 11.2% of inborn errors of metabolism in another pan-India tertiary care center study [26]. This systematic review provides a consolidated analysis of the existing literature on Gaucher disease among the Indian cohorts, emphasizing regional variations in mutations, clinical findings, diagnostic and therapeutic gaps, and mortality trends, and underscoring the need for context-specific strategies to manage Gaucher disease in India.

**Figure 3:**
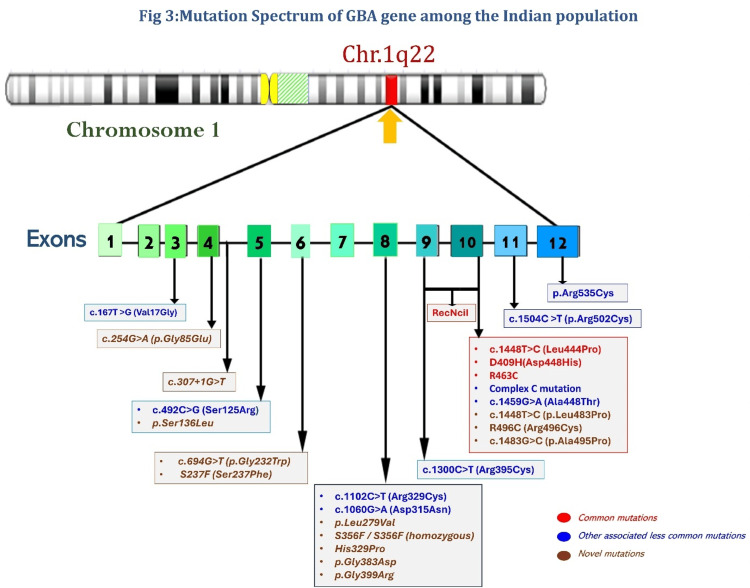
Mutation spectrum of the GBA1 gene reported in the Indian population. The figure has been modified from the original source by the first author and reproduced with courtesy of Barney et al. (2021) [25].

The genetic landscape of *GBA* mutations in the Indian population is diverse, encompassing both common global variants and several novel mutations. The p.Leu483Pro (L444P) mutation remains the most prevalent pathogenic variant observed, reported in 55% to 67% of Indian patients. Multiple studies consistently report its high frequency, with one large study involving 100 Indian patients identifying p.Leu483Pro in 62% of cases [25]. Another study detected the L444P variant in 60.60% of non-neuronopathic and 3.03% of sub-acute neuronopathic types among the 33 patients studied [21]. The p.Leu483Pro variant occurs in both homozygous and compound heterozygous forms (Figure 3) and is associated with variable phenotypic expression, spanning from non-neuronopathic type 1 Gaucher disease to neuronopathic type 3 Gaucher disease forms. The increased incidence of the p.Leu483Pro variant implies a common genetic origin or founder mutation for a substantial proportion of Gaucher disease cases across India.

In Indian Gaucher disease patients, the genotype-phenotype relationship largely mirrors global trends. Homozygous L444P mutations result in type 3 Gaucher disease with neurovisceral impact, whereas heterozygous mutations with one mild allele develop type 1 Gaucher disease with skeletal and visceral involvement without central nervous system signs. Subtle differences arise due to population-specific genetic modifiers and ecological variables, indicating that genotype alone may not reliably predict the precise clinical trajectory. Mutation patterns of Gaucher disease in India vary regionally, influenced by consanguinity and genetic drift, with a higher frequency of the p.Leu483Pro variant in the west. In some cases, a lack of mutation might suggest the possibility of large deletions/duplications or deep intronic variations, which are undetectable by conventional sequencing methods. These findings show the need for advanced genomic techniques, region-specific mutation databases, along with population-specific screening strategies to detect complex variants missed by standard sequencing to better delineate the genetic landscape of Gaucher disease in India [17].

Other frequently observed mutations include p.Asp448His (D409H), RecNcil, p.Arg329Cys, p.Arg502Cys, and p.Arg535Cys [27]. Notably, Indian researchers have also contributed to the global understanding of *GBA* mutations by detecting several novel missense mutations such as G289A(c.866G>C), I466S(c.1397T>G) (31), p.Ser136Leu, p.Leu279Val, p.His329Pro, c.307+1G>T, p.Gly383Asp, and p.Gly399Arg (16) (Figure 3).

Homozygous and compound heterozygous *GBA1* gene mutations are common, with many clustering around exons 8-11, signifying them as mutational hotspots. Regional distribution of GBA1 mutation across India is illustrated in the Appendices. Computational models envisage that novel variants such as G289A (c.866G>C) and I466S (c.1397T>G), located in the exons 7 and 10, respectively, destabilize the glucocerebrosidase protein structure by causing protein misfolding and loss of function [17]. A summary of genotypic variants in type I Gaucher disease in the Indian population is shown in the Appendices.

Gaucher’s disease exhibits a broad spectrum of clinical manifestations in Indian patients (Appendices). The non-neuronopathic form, type 1 Gaucher disease, was the most prevalent phenotype observed, reflecting global patterns. However, neuronopathic forms (types 2 and 3 Gaucher disease) are often reported with severe complications. Several studies have reported a significant overlap in phenotypic presentation between types 1 and 3, most often in relation to the pLeu483Pro (L444P) variant. Clinical symptoms typically begin in early childhood, including hepatosplenomegaly, anemia, thrombocytopenia, and skeletal abnormalities [27,31]. Diagnostic delay of up to 30 months persists due to low awareness, limited newborn screening, and low index of suspicion for rare diseases, worsening outcomes. In adults, the insidious onset and varied clinical presentation, similar to common hematological, liver, or bone disorders, complicates timely diagnosis [31].

Diagnostic confirmation in India follows a step-wise approach, beginning with dried blood spot (DBS) testing as the cost-effective primary screening option in India. However, the accuracy of DBS can be compromised by improper sample handling, exposure to high temperatures, or inadequate enzyme stability. Although β-glucocerebrosidase activity assays continue to remain the gold standard for diagnosis, plasma chitotriosidase enzyme activity has been widely used for clinical evaluation and monitoring since 1994 [32]. Gaucher cells release high levels of chitotriosidase, with elevated levels seen in untreated patients, making it useful for tracking therapeutic response [33]. Nevertheless, false negatives are observed in patients with *CHIT1* gene null alleles or those undergoing ERT. Other biochemical markers for Gaucher disease include CCL18, glucosylsphingosine, and ferritin. Glucosylsphingosine is a novel biomarker for Gaucher disease superior to both chitotriosidase and CCL18, making it valuable for patient monitoring [34]. Definitive diagnosis is made by the initial assessment of plasma chitotriosidase levels and subsequent confirmation by demonstrating reduced levels of β-glucocerebrosidase activity in leukocytes from peripheral blood [26]. Genetic analysis is confirmatory but frequently limited by affordability. Molecular characterization by bidirectional Sanger sequencing of the complete *GBA* gene coding region is crucial for pinpointing pathogenic mutations [21], confirming diagnosis, carrier screening, prenatal diagnosis, and genetic counseling of the family. Targeted screening in suspected pediatric cases with idiopathic splenomegaly and/or thrombocytopenia enhances early case detection [13].

ERT with recombinant glucocerebrosidase and SRT are recognized treatments for Gaucher disease, yet their affordability and access remain major hurdles. ERT works by replacing the deficient GCase with the recombinant GCase (imiglucerase). Imiglucerase replaces deficient GCase enzyme with mannose-exposed oligosaccharides, enabling uptake by macrophage mannose receptors and targeted delivery to the lysosomes for correction of the enzyme defect [1]. ERT improves hematologic parameters and visceral symptoms, particularly in patients with skeletal involvement [29]. With yearly treatment costs of INR 1.8-2.5 million per child and no public insurance coverage, most Indian patients rely mainly on pharma-sponsored charity programs for therapy. A 15-year retrospective analysis revealed 35% fatality within the Gaucher disease cohort, dropping to 10% with definitive treatment versus 47.5% with supportive therapy alone [27]. This striking difference highlights the importance of timely definitive treatment. Hematological parameters showed significant improvement even in low-dose ERT [18], but skeletal and visceral responses were limited with delayed or inconsistent therapy [18]. Baseline disease severity at presentation and genotypic profiles dictated therapeutic outcome.

SRT uses small-molecule drugs (e.g., miglustat) administered to decrease the synthesis of substances that amass in the disease. HSCT is a curative option in some cases, but is restricted by access and inherent risks. Survival data from Indian Gaucher disease patients reveal a reduced life expectancy, with early neurological decline in types 2 and 3 Gaucher disease, and poor outcomes in type 1 disease without timely treatment. Therapeutic interventions such as ERT or HSCT enhance survival substantially, while supportive care alone faced a sixfold higher risk of premature death.

Limitations

The small number of available studies, overlapping cohorts, and variability in outcome reporting precluded a formal meta-analysis; however, our systematic review provides a rigorous synthesis of the existing evidence and underscores the need for standardized, multicenter studies in the Indian population. Major limitations are retrospective study designs, often leading to recall bias, varying sample sizes, and small geographic coverage. Diagnostic method variations and molecular variability led to underreporting of certain complex *GBA* mutations [30]. The role of modifier genes such as *CHIT1* and *SMPD1* or environmental factors is underexplored in Indian studies, restricting deeper insights. Insufficient phenotypic data limit thorough genotype-phenotype correlation mapping. Tertiary care center studies tend to miss patients from underserved populations, distorting the overall clinical picture. Incomplete follow-up data often skew survival and therapeutic outcome data. These limitations reinforce the need for larger, multi-center prospective studies with standardized protocols for diagnosis, molecular characterization, and follow-up to offer a more complete and robust understanding of Gaucher disease in the Indian context.

## Conclusions

Gaucher disease poses a notable public health burden in India owing to its relatively high prevalence and poor treatment accessibility. Key challenges include low public awareness, limited diagnostic infrastructure, financial burden, and the lack of a national Gaucher disease registry to track disease impact and outcomes. Though India’s National Policy for Rare Diseases (2021) proposes financial support under the Rastriya Arogya Nidhi for HSCT, practical implementation remains inconsistent. Strengthening preventive strategies such as genetic counseling, prenatal diagnosis, and newborn screening is essential, particularly in communities with high consanguinity rates. By systematically consolidating the fragmented evidence, this review not only bridges a critical knowledge gap but also lays the groundwork for future research on genotype-phenotype correlations of Gaucher disease in the Indian population. A shift toward preventive community-based initiatives, establishing public-private collaboration, and regional lab empowerment is vital for reducing disease burden. Gaucher disease in India shows marked clinical heterogeneity, a distinct mutation pattern, dominated by the p.Leu483Pro variant with frequent diagnostic delays, and limited treatment access, often leading to poor prognosis. A targeted approach, including improved clinician education, accessible genetic diagnostics, and widened therapeutic coverage, is crucial to overcoming these hurdles. Future work must emphasize long-term treatment efficiency and the influence of modifier genes in disease progression.

## Figures and Tables

**Figure 4:**
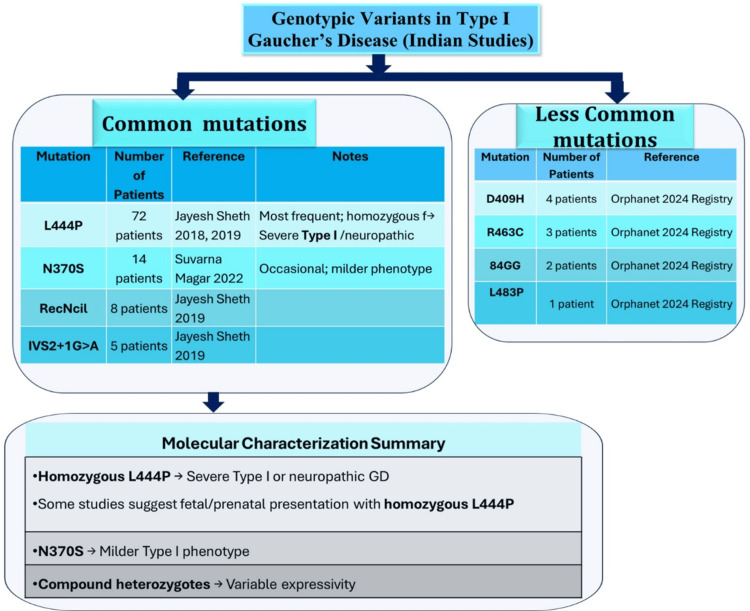
Summary of genotypic variants in type I Gaucher disease in the Indian population.

**Figure 5:**
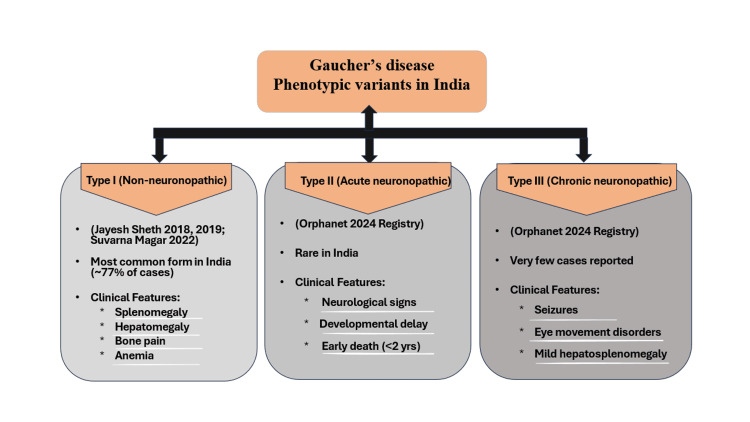
Summary of common phenotypic variants in Gaucher disease in the Indian population.

## Appendices

**Table 6:** Summary of the regional distribution of GBA1 mutations in India.

Region	Common mutations	Approximate frequency (%)	Notes
Northern India [24,28,30,31]	p.Leu483Pro (L444P), RecNciI, Complex C, p.Arg502Cys, p.Asp448His	Leu483Pro: ~60–67%	High mutation clustering; dominant in Delhi, Uttar Pradesh, Punjab; endogamy-driven frequency surge
		RecNciI: ~7–10%	
		Complex C: ~3–4%	
Western India [21,24,26,27,29-31]	p.Leu483Pro, p.Asp448His, p.Arg329Cys, p.Ser125Arg, p.Val17Gly, RecNciI	Leu483Pro: ~62%	Maharashtra shows a significant burden; studies suggest >1:600 carrier frequency
		Asp448His: ~6–7%	
Eastern India [24,31]	p.Leu483Pro, p.Ser356Phe, RecNciI, c.307+1G>T (splice-site), p.His329Pro	Leu483Pro: ~50–60%	Less studied, but includes several novel or rare variants with possible neurological involvement
		Novel variants: rare (<2%)	
Southern India [21,24,25,31]	p.Leu483Pro, p.Ala495Pro, p.Gly232Trp, p.Gly85Glu, p.Arg535Cys, RecNciI	Leu483Pro: ~55–60%	Scattered distribution; genetic testing is limited due to cost and access constraints
		Others: <5% each	

**Table 7:** Data extraction table. GD = Gaucher disease; LSD = lysosomal storage disorder; ERT = enzyme replacement therapy; SRT = substrate replacement therapy; HSCT = hematopoietic stem cell transplantation

Study identification						Population details									Diagnostic method			Gene mutation details							Clinical phenotype/Outcome variables								Treatment initiation (ERT/SRT)		Summary	Limitations	Statistical analysis	
Authors	Year of publication	Study title	Journal name	Country	Study design	Sample size	Total number of participants	Number of GD patients	Age range	Sex distribution	Ethnicity/Regions	Type 1	Type 2	Type 3	Enzyme assay	Gene sequencing	Both	N370S	L444P	Other mutations	Novel mutations	Zygosity	Mutation classification (pathogenic/likely pathogenic	Method of genotyping (PCR/sequencing/MLPA	Age of onset	Spleenomegaly (presence and severity)	Hepatomegaly (presence and severity)	Hematological findings (anemia (1), thrombocytopenia (2), leukopenia (3))	Bone involvement (bone pain (1), avascular necrosis (2), bone crisis (3))	Neurological involvement (eye movement disorder (1), seizures (2), cognitive decline (3))	Biomarker (chitotrioxidase/ferritin/Lysogb1/glycosyngo)	Disease severity (Simran score/GD-DS3/other clinical scores)	ERT/SRT	Response to treatment (improvement in symptoms/biomarkers)				
Sheth et al. [31]	2019	Gaucher disease: single gene molecular characterization of one-hundred Indian patients reveals novel variants and the most prevalent mutation	BMC Medical Genetics	India	An observational molecular characterization study	100	100 (includes 90 children, 8 adults, 2 fetal samples)	100	1 day to 31 years	62 males/36 females/2 fetuses	Predominantly Western India (43%), Northern (32%), Southern (21%), Eastern (4%)	77	12	11	Yes (β-glucosidase activity and chitotriosidase)	Yes (Sanger sequencing of the full *GBA1* coding region)	Yes	No	p.Leu483Pro (L444P): most prevalent (62%)	p.Arg535Cys (~7% ), RecNcil(≈7%), complex C mutation (3%)	4 novel missense mutations: p.Ser136Leu, p.Leu279Val, p.Gly383Asp, p.Gly399Arg	71 homozygous; 22 compound heterozygous; 4 proband-parents tested posthumously; 3 undetermined	All novel mutations were predicted to be pathogenic/disease-causing via in silico analysis	PCR-RFLP for initial screening; nested PCR and Sanger sequencing for the full *GBA1* gene; MPLA not mentioned	1 day to 31 years. Precise onset not detailed per patient, but neurological regression was noted from age 1.5 years to 8 years in some GD2/GD3 cases	Present in all patients. Described as moderate to severe	Present in most patients. Mild to severe hepatomegaly documented	1: Present in all or most patients. 2: Present. 3: Not explicitly reported	1-YES Mentioned generally (not quantified),2-NO,3-NO	1: No. 2: Yes (mentioned as part of the GD2/GD3 spectrum). 3: Yes (neurological regression mentioned in GD2 and GD3 patients)	Chitotriosidase: Elevated in 71 patients (range: 172–72,000 nmol/h/mL), normal in 13, and undetectable in 5. β-Glucosidase activity was reduced in nearly all patients (diagnostic tool). Others: No	Not used	ERT: Given to only 2 patients (due to cost constraints). SRT: No	Not reported or assessed in this study due to limited therapy access and lack of longitudinal follow-up data	GD type distribution: Type 1 (77%), Type 2 (12%), Type 3 (11%). Most common mutation: p.Leu483Pro (L444P) – seen in 62% of patients. Other common mutations: p.Arg535Cys and complex allele RecNcil (each ~7%). Novel mutations: Identified 4 novel missense mutations – p.Ser136Leu, p.Leu279Val, p.Gly383Asp, p.Gly399Arg. Enzyme deficiency: All had significantly low β-glucosidase levels. Chitotriosidase: Elevated in 71% of patients. Geographic clustering: Predominantly Western and Northern India. Zygosity: 71 homozygous; 22 compound heterozygous; 3 undetected cases. Consanguinity: Found in 26% of cases	Lack of longitudinal follow-up: Treatment response (e.g., to ERT) not systematically evaluated. Only 2 patients received ERT due to cost constraints. Absence of clinical severity scoring: No validated disease severity tools (e.g., GD-DS3, Simran Score) were used to assess clinical progression or compare genotype-phenotype. Limited functional validation of novel mutations: Novel mutations were identified using in silico tools only; no wet-lab functional assays were performed. No genomic structural analysis: In 3 patients, mutations were not detected; the authors hypothesize large deletions/deep intronic variants, but MLPA or WGS was not performed. Sample bias: Patients were referred through specific centers in India, predominantly from western and northern regions – possibly limiting generalizability to all Indian subpopulations. No statistical testing of genotype-phenotype correlations: While patterns were observed, no formal statistical analysis was performed to test associations between specific mutations and clinical features	Descriptive statistics used: Frequencies/Percentages for mutation prevalence, GD types, regional distribution, and gender distribution. Ranges: Age (1 day to 31 years). Chitotriosidase activity (172–72,000 nmol/h/mL). No use of statistical comparisons or inferential analysis. In silico prediction tools were used (e.g., MutationTaster, SIFT, PolyPhen-2) for functional prediction of novel mutations	
Magar et al. [26]	2022	Targeted screening for Gaucher disease in high suspicion patients and clinical profile of screen positives in a large pediatric multispecialty hospital	Cureus	India (MGM Institute of Health Sciences, Aurangabad, Maharashtra)	A prospective observational study (January 2020 to September 2022)	22 pediatric patients	22 pediatric patients	9 (40.9%)	Birth to 18months	6 males, 3 females	Indian pediatric population in Western India from a large multispecialty hospital in Maharashtra, India	No	No	No	Yes: Enzyme glucocerebrosidase (2.3-14.1 nmol/mL/hr) and chitotriosidase levels (DBS, N < 200 nmol/mL/hr) were done for 6 patients	Yes: DNA mutation analysis (gene sequencing) for 3patients	Yes (6 patients)	No	p.Leu483Pro-most prevalent: 6 patients		c.254G>A p.Gly85Glu homozygous,c.694G>T p.Gly232Trp homozygous, c.1504C>T (p.Arg502Cys) homozygous, c.1448T>C (p.Leu483Pro) c.1483G>C (p.Ala495Pro) Compound heterozygous	8 homozygous; 1 compound heterozygous	No	DNA mutation analysis (likely Sanger sequencing)	Birth to 18 months	Yes: Moderate to massive splenomegaly - 8 patients, mild splenomegaly - 1 patient	Yes: Mild to moderate hepatomegaly in all patients; the neonate exhibited only mild enlargement	1: Present in 8 patients. 2: Present in 6 patients. 3: Present in 5 patients. Yes: Packed cells and platelet transfusions - 1 patient, packed cell transfusion - 4 patients	Yes: Spinal fracture. 1: No. 2: No. 3: No. Femur fracture - 1 patient	1: No. 2: Yes (yes, multiple seizures - 1 patient, 3 -developmental delay) - 1 patient	Hemoglobin, platelets, liver size monitored, others - no	No	ERT: Imiglucerase used for 1 patient. SRT: No	Notable increase in hemoglobin and platelets, reduction in liver size, improved well-being after 2 months	Feature details participants: 22 screened; 9 confirmed. GD diagnosis method: 6 by enzyme assay, 3 by gene sequencing. Gene mutation: p.Leu483Pro (L444P); N370S absent. Clinical findings: Hepatomegaly (all); anemia, thrombocytopenia; transfusions in 5 cases. Bone/Neuro/Biomarkers: Not documented. Severity score: Not applied. ERT administered: 1 patient (imiglucerase, 2 months). Response to ERT: Hb/platelet recovery, reduced liver size, clinical improvement	Small sample size: Only 22 high-suspicion pediatric patients were screened. The limited number of diagnosed GD patients (n = 9) reduces the generalizability of findings. Single-center study: Conducted at one tertiary care center (MGM Hospital, Aurangabad), which may not reflect broader epidemiological trends. Short follow-up: Only one patient received ERT, and follow-up was limited to two months, which restricts long-term outcome assessment. Limited genetic data: Gene sequencing performed in only 3 patients; mutation profiles may therefore be underrepresented. No zygosity or comprehensive mutation classification was provided. Lack of biomarker evaluation: Important Gaucher biomarkers such as chitotriosidase, ferritin, and Lyso-Gb1 were not measured due to infrastructure limitations. No standardized severity score used: Clinical severity was not assessed using tools like GD-DS3 or Simran score	Descriptive statistics only: The analysis was purely descriptive. Data were presented as: frequencies (e.g., number of patients with hepatomegaly, anemia, etc.), percentages (e.g., 40.9% diagnosed with GD), means, and/or ranges (though specific ranges were not detailed). No inferential statistics applied: No hypothesis testing (e.g., t-tests, chi-square) or confidence intervals were used. No multivariate analysis or regression modeling was conducted	
Sheth et al. [30]	2018	Biochemical and molecular characterization of adult patients with type I Gaucher disease and carrier frequency analysis of Leu444Pro – a common Gaucher disease mutation in India	BMC Medical Genetics	India	An observational study	1207	7 adult GD patients + 1,200 population subjects (for carrier frequency)	7	20–40 years	3 males/4 females	Gujarat, Punjab, Uttar Pradesh, Maharashtra (Western and Northern)	Yes: All 7 patients had type I GD	NO	No	Yes: β-glucosidase activity measured from leukocytes	Yes: Full *GBA* gene via Sanger sequencing	Yes	No	Found in 3/7 patients (heterozygous)	Arg329Cys, Asp315Asn, Ser125Arg, Arg395Cys	Ala448Thr, Val17Gly	4 homozygous, 3 compound heterozygous	Pathogenic/likely pathogenic (in silico + structural modeling)	PCR-RFLP for L444P; Sanger sequencing for the full *GBA* gene	Adulthood (20–40 years); childhood normal	Present in all patients. Moderate to severe	Present in 3/7 patients	1: Present in 4 patients. 2: Present in 4 patients. 3: Cytopenia in at least 1 patient	1: No. 2: Yes (1 patient). 3: No. Sclerosis and MRI changes in others. Gaucher cells were seen in 4/7 cases	No (not seen in type 1)	β-Glucosidase activity: Deficient (<10%) in all patients. Chitotriosidase: Elevated in 5 patients (1670 to 72,000 nmol/h/mL), normal in 1, undetectable in 1. Ferritin/Lyso-Gb1/Glycosyngolipid: Not measured	NO	ERT: Given to 2 patients (imiglucerase) before molecular diagnosis. SRT: Not mentioned	Improvement in chitotriosidase was not specifically measured, but the literature cited decreased chitotriosidase with ERT; low activity was seen in treated patients (possibly due to therapy)	All 7 patients had type I GD. Splenomegaly was universal; hepatomegaly in 3; cytopenias (anemia, thrombocytopenia) were common. Bone abnormalities (e.g., avascular necrosis) were present in some. Chitotriosidase was elevated in 5/7; normal/undetectable in 2 (possibly due to *CHIT1* null allele or ERT). All patients had <10% of normal β-glucosidase activity. Mutations identified: Common: Leu444Pro (heterozygous in 3 patients). Two novel mutations: Ala448Thr and Val17Gly. Zygosity: 4 homozygous; 3 compound heterozygous. Carrier frequency of Leu444Pro: 1 in 600 in the general Indian population	Small patient cohort: Only 7 adult GD patients studied, which limits generalizability and statistical power. No neurological evaluation: Neurological aspects were not explored; only confirmed absence of symptoms. Incomplete data for 1 patient. Clinical history of patient P4 was unavailable. ERT response was not evaluated. Although 2 patients were on ERT, clinical/biomarker response was not longitudinally assessed. No disease severity scoring system was used. Tools such as GD-DS3 or Simran score were not applied. Novel mutations were not functionally validated. Functional validation was in silico only – no in vitro or cellular assays done. Geographic limitation: The majority of cases were from Western India, so findings may not be representative of all Indian regions. Single-mutation carrier screening. Only Leu444Pro was screened in the population; other pathogenic mutations may have been missed	Analysis performed: Detailed descriptive statistics such as age, sex, clinical features, enzyme activity, mutation frequency, and allele and genotype frequencies calculated via the Hardy-Weinberg formula. Sample size justification: Used an online sample size calculator for general population screening. In silico pathogenicity: Six different software tools were used to predict novel mutation effects. No inferential statistics: No hypothesis testing (e.g., chi-square, t-tests) or regression was used	
Sheth et al. [24]	2024	Burden of rare genetic disorders in India: twenty-two years’ experience of a tertiary centre	Orphanet Journal of Rare Diseases	India	A retrospective observational registry-based study	6,245 patients with rare genetic disorders	6,245 patients with rare genetic disorders	253 patients with GD	Pediatric to adult. Median age of diagnosis: 3.5 years for LSDs	Not explicitly provided for GD patients	Indian population, national referral center	Type 1 (most common)	Yes, but not quantified	Yes, but not quantified	Yes: β-glucocerebrosidase assay used in diagnosis	Yes: *GBA1* sequencing used in a subset of cases	Yes: Confirmatory diagnosis was used for many patients	14 patients (5.5%)	107 patients (42.3%)	D409H-30 patients (11.9%), RecNciI 24 patients (9.5%), R463C-18 patients (7.1%)		Partial: Some genotype combinations, such as homozygous L444P, were mentioned. Full breakdown not provided	Mutations were described as pathogenic	Sequencing (likely Sanger), no mention of MLPA or PCR separately	Median age: ~3.5 years	Outcomes (O)	Partial	Basic phenotype mentioned; no severity scoring or clinical follow-up	1: Yes. 2: No. 3: No	1: Yes. 2: Yes. 3: Yes (in neuronopathic GD (types 2/3))	No mention of chitotriosidase, ferritin, Lyso-Gb1	Not used	Yes: Many GD patients were on ERT; no specifics on SRT	Yes: Clinical improvement noted in treated patients (e.g., reduction in spleen size, hematological recovery), though not quantitatively assessed in detail	Key findings: A total of 6,245 patients with suspected genetic disorders were referred; 1,727 cases (27.6%) were diagnosed with LSDs. Among LSDs, 253 patients (14.6%) were diagnosed with GD. Common mutations identified in Gaucher disease: L444P (42.3%); D409H (11.9%); RecNciI (9.5%); R463C (7.1%); N370S/N409S (5.5%). Gaucher disease was more common than other LSDs in the cohort. Diagnostic methods included enzyme assays, tandem mass spectrometry, and confirmatory genetic testing for *GBA1* mutations. A substantial delay between the onset of symptoms and diagnosis was noted in many cases	Retrospective design: Data were collected from existing medical records, which may have gaps or inconsistencies. Incomplete genetic testing: Not all patients underwent full gene sequencing; some were diagnosed based only on enzyme activity. Lack of clinical outcome data: The study does not comprehensively report clinical progression, treatment responses, or survival outcomes. No severity scoring system: Clinical severity scores (like GD-DS3 or Simran score) were not used. Limited phenotypic classification: Specific GD subtypes (types 1, 2, 3) were not quantified or analyzed separately. Missing treatment response data: Efficacy of ERT or SRT is not statistically evaluated	The study was primarily descriptive. Data presented using counts and percentages for disease types and mutation frequencies. Median age of diagnosis was mentioned for the LSD group. No inferential statistical tests (e.g., chi-square, t-test, regression) were performed. No p-values or confidence intervals were reported	
Muranjan and Patil [29]	2016	Outcome of Gaucher Disease in India: Lessons from Prevalent Diagnostic and Therapeutic Practices	Indian Pediatrics	India	A retrospective and prospective observational study	37	37	37 (confirmed cases)	Onset: 1–150 months. Diagnosis: 4–229 months	22 males/15 females; M:F = 1.46:1	Indian children, single-center from Western India (Mumbai)	45.4% (10/22 with phenotypic classification)	Not clearly mentioned (likely minimal or none)	41% (9/22)	Yes: β-glucocerebrosidase activity used for diagnosis	Yes: Mutation analysis performed (subgroup of patients). Sanger sequencing (via expert centers)	Yes: Used for confirmed diagnosis in many cases	Not detected	Detected in 10 patients (62.5%): 8 homozygous, 2 heterozygous			Homozygous (8). Heterozygous (2) for Leu483Pro	Pathogenic (Leu483Pro)	Sanger sequencing (via expert centers)	Mean: 28 months (range: 1–150 months)	Present in all; severe in 9/9 with intact spleen (average volume 40.2x normal)	Present in all before ERT; 4 moderate, 4 severe; avg liver volume: 2.27x normal	Anemia: 83.7% (most severe in 46%). Thrombocytopenia: 56.7%. Leukopenia: 32.4%	Osteopenia in 75%; suboptimal skeletal response; BMD improvement in 22%	Type 3: Eye movement disorders (e.g., horizontal gaze palsy), seizures, cognitive decline (not detailed for all)	Plasma chitotriosidase was elevated in 85.7% (9 had >15,000)	Gaucher Severity Score Index (GauSSI)	ERT (imiglucerase); SRT not mentioned	Partial response; hematologic parameters improved early; visceral and bone outcomes suboptimal due to late onset and low-dose ERT	This single-center retrospective and prospective observational study analyzed 37 patients with confirmed GD treated at a tertiary care center in Mumbai, India, from January 1995 to June 2013. The aim was to assess disease severity and response to ERT using the GauSSI and recommended therapeutic goals. Key observations: Mean age of onset: 28 months. Delayed diagnosis: Average lag of 30 months. Phenotypic classification: 45.4% type 1, 41% type 3. Common mutation: Leu483Pro (L444P) – 62.5% (mostly homozygous). ERT was initiated in 12 cases (Imiglucerase via the INCAP program). Partial therapeutic response was achieved in hematological domains, but suboptimal outcomes in visceral and skeletal domains due to low dosing and late initiation	Retrospective design: A significant portion of data was collected retrospectively (83.8%), which limits the completeness and consistency of clinical and biochemical records. 2. Incomplete clinical evaluations: Due to financial constraints, full imaging assessments (e.g., for organomegaly, bone, lungs) were not performed in all patients. 3. Loss to follow-up: Some patients were lost to follow-up, reducing long-term outcome evaluation. 4. Short duration of ERT: The majority had ERT for <3 years, which limits conclusions on long-term skeletal/growth responses that require >3 years of treatment. 5. No control group: The study lacked a comparator group (e.g., untreated patients or those on standard-dose ERT). 6. Small sample size for ERT response analysis: Only 11 patients were available for prospective ERT response analysis. 7. Variable dosing of ERT: Due to supply limitations, patients received suboptimal or varied doses, making it difficult to correlate outcomes strictly with treatment	Descriptive statistics (means, ranges, percentages): Age of onset, diagnosis, and treatment duration. Proportion of patients achieving therapeutic goals. Severity grading using GauSSI score (for anemia, thrombocytopenia, hepatomegaly, splenomegaly, chitotriosidase). Clinical response assessment using published therapeutic goal timelines for hemoglobin, platelet count, liver/spleen volume, BMD, and growth. Tabular presentation comparing pre- and post-ERT status across clinical domains	
Ankleshwaria et al. [21]	2014	Novel mutations in the glucocerebrosidase gene of Indian patients with Gaucher disease	Journal of Human Genetics	India	A cross-sectional, molecular genetic analysis	45 unrelated patients with Gaucher disease	45	45	Pediatric age group, mostly; specific range not given	Not specified	Indian patients from various regions (multi-centric collaboration)	Primarily type I	Not detailed	Not detailed	Yes: Used for initial diagnosis	Yes: Full *GBA1* sequencing performed	Yes: Confirmatory diagnosis involved both tests	yes	L444P (most common)	D409H, R463C; 7 novel mutations identified		Both homozygous and compound heterozygous mutations were reported	Described using in silico tools and evolutionary conservation, categorized as pathogenic/likely pathogenic	Sanger sequencing (PCR-based amplification and sequencing)	Not clearly defined per patient; the study focuses on genotype data	Yes (but not quantified)	Yes	1: Yes. 2: Yes. 3: Yes (but not detailed per individual case)	1: No. 2: No. 3: No	1: No. 2: No. 3: No	Not specified	Not used	No	Not assessed – this study focused on mutation discovery	Objective: To identify known and novel mutations in the GBA1 gene of Indian patients with GD and expand the mutational spectrum relevant to this population. Design: A cross-sectional molecular study of 45 unrelated Indian patients diagnosed with GD (primarily Type 1) based on clinical findings and β-glucocerebrosidase enzyme assay. Methods: DNA extracted from patients was subjected to PCR amplification and Sanger sequencing of the entire GBA1 coding region. Bioinformatics tools (e.g., PolyPhen2, SIFT) were used to assess the pathogenicity of novel variants. Findings: A total of 16 different mutations were identified. 7 novel mutations were reported, including missense, nonsense, and small deletion variants. L444P was the most frequent known mutation. Zygosity analysis showed both homozygous and compound heterozygous states. The study contributes to understanding GD's genetic heterogeneity in the Indian population, which is critical for diagnosis, carrier detection, and counseling.	Lack of clinical correlation: No detailed correlation was made between specific mutations and the clinical severity (e.g., organomegaly, bone crisis, neurological status). No longitudinal data: The study was not designed to follow patients over time or assess treatment response or prognosis. No biochemical or biomarker analysis: Biomarkers such as chitotriosidase, ferritin, or Lyso-Gb1 were not measured. Small sample size: The study included only 45 patients, which limits the statistical power to detect associations between genotype and phenotype. Single-timepoint assessment: All data were based on initial diagnosis; age of onset, disease progression, or treatment outcomes were not captured	The study was primarily descriptive and exploratory. No formal statistical analysis (e.g., chi-square tests, p-values, confidence intervals) was performed. Bioinformatic prediction tools (e.g., PolyPhen2, SIFT) were used to assess the pathogenic potential of novel mutations, which is computational rather than inferential. Mutation frequencies were reported as counts and percentages	
Barney et al. [25]	2021	Clinicogenetic Profile, Treatment Modalities, and Mortality Predictors of Gaucher Disease: A 15-Year Retrospective Study	Public Health Genomics	India	A retrospective cohort study	60 patients with Gaucher disease	60 patients with Gaucher disease	60 (all types)	0.7 to 12.5 years	31 males (51.7%)/29 females (M:F ≈ 1.07:1)	Mostly South and North India (6 from Bangladesh)	Majority (exact number not explicitly stated; all HSCT cases were Type I) - 48 patients	2 patients (3.3%)	10 patients (16.7%)	β-glucocerebrosidase: Yes – in 53/60 (88%), all low. Bone marrow biopsy: Yes – 80% (showed Gaucher cells)	Yes: In 29/60 (48%)	Performed in 29 patients	Not reported	Found in 20/29 (67.8%) patients (homozygous)	Asp448His (D409H), Arg502Cys, His329Pro (novel), c.307+1G>T (novel splice-site)		26 homozygous, 3 compound heterozygous	All pathogenic or likely pathogenic (in silico validation used)	Sanger sequencing, confirmed with HGMD annotations	2 years (IQR = 1–9 years)	Not specified	Not specified	1: No. 2: No. 3: No	1: 5/60 (8.3%). 2: No. 3: No	1: 4/60 (6.6%). 2: 9/60 (15%). 3: 20/60 (33.3%)	Chitotriosidase: Available in 21 patients; elevated, but not significantly predictive of mortality. Enzyme Level (β-glucocerebrosidase): Yes (mean: ~1.7 nmol/h/mL (reference: 2.3–18.4)). Ferritin/Lyso-Gb1/Others: No	GD-DS3/Simran score: Not applied. Mortality risk predictors: Based on regression analysis	ERT-9 patients SRT-Included in 8 patients (clinical trial) HSCT-3 patients (Type I GD, post-transplant follow-up up to 10 years) Supportive Therapy-40 patients (66.7%)	Improved Hb, platelet counts, and organ size in ERT/HSCT patients. Mortality Rate: 35% overall; 10% in definitively treated vs. 47.5% in supportive therapy group. Kaplan-Meier survival: Significant difference (p = 0.001) between treated vs. supportive	To describe the clinical and genetic features, treatment modalities, and predictors of mortality in Gaucher disease (GD) patients evaluated over 15 years at a tertiary pediatric center in India. Study design: A retrospective cohort study. Period: 2003–2018. Setting: Christian Medical College, Vellore. Participants: 60 children diagnosed with Gaucher disease. Key findings: Median age at diagnosis: 2 years. 20% had neuronopathic GD (types 2 and 3). Common clinical features: hepatosplenomegaly (98.3%), anemia (71.6%), thrombocytopenia (87%). Genetic testing was done in 29 patients: L444P homozygosity was the most common (67.8%). A 35% mortality rate was observed. Mortality was significantly higher in patients who received only supportive therapy. Treatment (ERT, SRT, HSCT) improved survival and clinical outcomes	Retrospective single-center study: Limits generalizability; subject to documentation bias. Incomplete genetic data: Only 48% underwent molecular testing due to cost and accessibility. Lack of standardized clinical scoring systems. Disease severity scores (e.g., GD-DS3) were not applied. Limited biomarker data. Chitotriosidase levels were measured in only 21 patients; other markers such as Lyso-Gb1 were not used. Small treated cohort. Only 9 received ERT and 3 HSCT; limited power for subgroup comparisons. No long-term quality-of-life data. Functional outcomes and neurocognitive progress were not systematically captured post-treatment	Descriptive statistics: Mean, median, IQR for continuous variables; frequency and percentage for categorical data. Kaplan-Meier survival analysis: Used to estimate survival; significant difference (p = 0.001) between treated vs. supportive care groups. Univariate analysis: Fisher’s exact test and Mann–Whitney U test used to identify mortality predictors. Multivariate regression: Not performed (likely due to limited sample size). Software used: Not specified, but standard statistical methods were used	
																																						Verma et al. [28]	2012	Spectrum of Lysosomal Storage Disorders at a Medical Genetics Center in Northern India	Indian Pediatrics	India	A descriptive (retrospective observational study)	68 confirmed LSD patients	93 suspected LSDs	10	5 months to 26 years (mean 4.5 years)	76.4% male overall for LSDs (specific M/F for GD not detailed)	North India	8 (non-neuronopathic)	1 (acute neuronopathic)	1 (sub-acute neuronopathic)	Yes (blood leukocyte and/or skin fibroblast)-β-glucosidase activity (nmol/h/mg) and ranged from 0.38–3 in blood; 10–21 in skin fibroblasts	Yes (mutation data available for 3 families)	Yes, in some cases	No	Found homozygous in 1 family	S237F/R496C (compound heterozygous); S356F/S356F		1 homozygous (L444P), 2 compound heterozygous	Pathogenic	Whole gene sequencing (outsourced to international labs)	Childhood (specific age not detailed)	Yes in all patients	Yes in all patients	1: Yes (8/8). 2: Yes (8/8). 3: No	1: Yes. 2: No. 3: Yes	Present in GD types 2 (1 patient) and 3 (1 patient) (no specific features reported)	β-glucosidase enzyme activity measured in blood/fibroblasts. Chitotriosidase: Not reported. Ferritin Lyso-Gb1: Not measured	Not used	ERT: Given to 4 patients with type 1 GD. SRT: Not used	Improvement in growth, hemoglobin, platelets, and liver/spleen size; no adverse events reported	Objective: To describe the clinical, biochemical, enzymatic, and molecular characteristics of patients diagnosed with lysosomal storage disorders (LSDs) at a tertiary medical genetics center in North India over a 3-year period (2008–2010). Study design: Descriptive, retrospective observational study. Participants: 93 suspected LSD cases, 68 patients confirmed with LSDs based on enzyme assays ± genetic studies. Key findings: GD was diagnosed in 10/68 confirmed cases: Type 1: 8 patients; type 2: 1 patient; type 3: 1 patient. Most common clinical features (across LSDs): Growth retardation (47.2%); hepatosplenomegaly (41.2%); neuroregression (33.8%). Consanguinity in 32.4% of families. Molecular diagnosis was possible in 7 families, including 3 with Gaucher mutations: L444P/L444P, S356F/S356F, S237F/and R496C. ERT was administered to 8 patients, including 4 with GD, who showed clinical improvement in hematologic and visceral symptoms. Prenatal diagnosis performed in 6 families (including 1 Gaucher case)	Small sample size per LSD subtype: Only 10 Gaucher disease patients, limiting statistical power and subgroup analysis. Lack of systematic neurological phenotyping. Features such as seizures, cognitive decline, and oculomotor dysfunction were not quantified, even in neuronopathic GD cases. Incomplete molecular analysis-Genetic testing was available in only 7 families due to financial/logistical constraints. Single-center, referral bias. All patients were from a North Indian tertiary care center, which may not reflect national diversity. No use of standardized clinical severity scores. Tools like GD-DS3, Simran score, or quality-of-life scales were not applied. Limited longitudinal follow-up. Only early response to ERT was reported; long-term outcomes were not evaluated. Absence of biomarker data (e.g., Lyso-Gb1, chitotriosidase). Biochemical markers were not assessed or reported beyond enzyme activity	Descriptive statistics: Frequencies, percentages, and enzyme activity ranges. Quantitative data: Enzyme levels (e.g., β-glucosidase 0.38–3 nmol/h/mg). Mutation prevalence: Reported descriptively (e.g., L444P in 1 family). No inferential statistics: No hypothesis testing (no p-values, no multivariate analysis). Software used: Not mentioned		
																																																																												Goyal et al. [27]	2021	Lysosomal Storage Disorders: Clinical, Biochemical and Molecular Profile from Rare Disease Centre, India	Annals of Indian Academy of Neurology	India	A retrospective observational study	65 children with LSDs	65	30	6 months to 13 years	26 males/4 females (M:F = 6.5:1)	Rajasthan	The majority (not explicitly distinguished, but all had hepatosplenomegaly and cytopenia	Not clearly differentiated	Not clearly differentiated	Yes: β-glucocerebrosidase (activity <10% of normal)	Yes: In 14 patients	Yes (for 14 patients)	Not detected	Detected in 12 of 14 genetically analyzed patients (most were homozygous)	Compound heterozygous with RecNcil and one with R496C		Majority homozygous: 13 patients; some compound heterozygous: 1 patient	Pathogenic	Sanger sequencing (molecular lab referral)	Infant to early childhood (11 months to 13 years)	All 30 cases showed splenomegaly	All 30 cases showed hepatomegaly	1: Yes. 2: Yes. 3: Yes (bicytopenia (anemia + thrombocytopenia): 14/30 = 46.7% anemia alone: 9/30 = 30.0% pancytopenia (anemia + thrombocytopenia + leukopenia): 7/30 = 23.3%)	1: No. 2: No. 3: Yes (one patient had osteomyelitis and bone crisis; Erlenmeyer flask deformities mentioned)	1: No. 2: No. 3: No	Chitotriosidase- Used for screening GD and NP disease (results not numerically detailed). Ferritin/Lyso-Gb1/Others: Not reported	Not used	ERT: Given to 2 GD patients via compassionate access program. SRT: Not used	Improvement in hemoglobin, platelets, liver/spleen size, and physical activity noted; no serious side effects observed	Study type: Retrospective observational study. Study period: December 2016 to December 2019. Setting: JK Lon Hospital, SMS Medical College, Jaipur. Population: 65 children diagnosed with LSDs. Data collected: Clinical features, family history, enzyme assay results, genetic testing (when feasible), treatment, and follow-up. Key findings: GD was the most common LSD (46.1%), followed by MPS (35.3%). GD was more common in males (26/30); age range 6 months to 13 years. Common GD features: hepatosplenomegaly, anemia, thrombocytopenia, and bone involvement (including osteomyelitis and crisis). Enzyme assay confirmed GD with β-glucocerebrosidase activity <10% in most patients. Molecular testing was performed in 14 GD cases; L444P mutation - 12 cases, including compound heterozygotes with RecNcil. ERT was initiated in 2 GD patients & showed clinical improvement in hematologic and visceral parameters. Emphasis was placed on the importance of early diagnosis, genetic counseling, and prenatal screening	Single-Center study: Data limited to one institution in Rajasthan; findings may not be generalizable across India. Referral bias: The majority of referred cases had strong clinical suspicion; underdiagnosis in other populations was possible. Incomplete genetic testing: Molecular diagnosis was only performed in 14/30 GD patients due to cost/logistics constraints. Small treated cohort: Only 2 GD patients received ERT, limiting insights into long-term treatment response. Lack of severity scoring: Disease severity tools like GD-DS3 or Simran Score were not applied, reducing the standardization of clinical assessment. No control/comparator group: The study lacks a non-LSD comparator group or inter-group comparisons (e.g., between GD and MPS). Limited biomarker panel: Biomarkers like Lyso-Gb1, ferritin, or glucosylsphingosine were not assessed. Limited neurological evaluation: Neurological involvement in neuronopathic GD types was not systematically reported.	Descriptive Statistics: Used throughout for age, sex, organ involvement, and mutation frequencies. Frequencies/Percentages: For clinical features, LSD subtype distribution, and treatment count. No inferential statistics: No p-values, confidence intervals, regression models, or correlation analyses. Comparative analysis: Not performed between subgroups (e.g., treated vs untreated GD cases). Mutation reporting: Genotypic frequency summarized descriptively (e.g., 12/14 had L444P mutation)		
